# Recommendations for appropriate sublingual immunotherapy clinical trials

**DOI:** 10.1186/1939-4551-7-21

**Published:** 2014-10-06

**Authors:** Giovanni Passalacqua

**Affiliations:** Allergy and Respiratory Diseases, IRCCS S.Martino Hospital – IST – University of Genoa, L.go R Benzi 10, Padiglione Maragliano, 16132 Genoa Italy

**Keywords:** Sublingual Immunotherapy, World Allergy Organization (WAO), Local Side Effects, Culprit Allergen, Peak Pollen Period

## Abstract

Sublingual immunotherapy is currently considered a viable alternative to the subcutaneous route. The body of evidence of its efficacy is based on the results of 77 clinical trials and 7 meta-analyses, that have been published so far. Nonetheless, the experimental evidence is partially weak due to the large heterogeneity of studies, namely: doses, regimens, patient selection, duration of treatment, outcomes and reporting. In addition, it is virtually impossible to compare the potency of extracts produced by different manufacturers. Also, there is large variability in reporting and in the classification of adverse events, either systemic or local, so that only a rough estimate can be provided. Considering all these aspects, efforts are needed to harmonize the methodology, outcome measures and reporting of SLIT clinical trials, to achieve the ability of comparing the results of various studies. International societies and the World Allergy Organization have recently provided general recommendations on how to design and conduct trials which can provide more interpretable and homogeneous data.

## Introduction and background

The subcutaneous modality of specific immunotherapy (SCIT) remained for many decades the only available route of administration for allergen immunotherapy. SCIT is effective and safe, when properly prescribed and administered, but a certain level of risk of severe side effects still remains [1]. The problem of the risk/benefit ratio prompted, mainly during the 1980’s, the search for safer routes of administration, among which, the sublingual one (SLIT), first described in 1986, achieved both scientific and clinical importance. In less than 25 years since the first report, SLIT gained credibility, and was therefore introduced in the official documents as a viable alternative to the classic injection route [2, 3] for both adults and children. Presently, SLIT is commercialized and routinely used in Europe and in many other countries, and some products have also been approved recently in the USA.

According to the more recent World Allergy Organization (WAO) Position Paper on SLIT [4], there are 77 randomized placebo controlled trials published, of which only 8 have demonstrated negative results. Some studies have clearly shown a dose-dependent effect of SLIT [5, 6], indicating robust support in favor of its clinical efficacy. Due to the large number of trials available, several meta-analyses have been carried out (Table 1), with various inclusion criteria such as rhinitis and asthma [7, 8], asthma only [9], asthma and/or rhinitis in children [10, 11], conjunctivitis [12]. Moreover, separate meta-analyses for single allergens such as dust mite [13] or grasses [14] have also been published, with favorable results as well. On the other hand, the validity of the meta-analyses remains limited by the high degree of statistical heterogeneity as a result of the heterogeneity of the studies themselves. Main sources of heterogeneity are: criteria of inclusion, sample size, duration of treatment, administration regimens, maintenance dose, standardization of extracts, outcomes, reporting, and the description of adverse events. Table 2 evidences the main variability aspects. All these elements make the trials difficult to compare.

**Table 1:** **Meta analyses of slit randomized controlled trials**

Author	Population	Disease*	Active/Placebo	SMD**	Heterogeneity I^2^
Wilson et al. [7]	Adults + Children	R	484/475	-0.42	73%
Penagos et al. [10]	Children < 18 y	R	245/239	-0.56	81%
Calamita et al. [9]	Adults + children	A	150/153	-0.38	64%
Penagos et al. [11]	Children < 18 y	A	232/209	-1.14	92%
Compalati et al. [13]	Adults + children only mite	RAC	194/188	-0.95	92%
Di Bona et al. [14]	Adults + children only grass	RAC	1518/1453	-0.32	56%
Radulovic et al. [8]	Adults + children	R	2333/2256	-0.49	81%
Calderon et al. [12]	Adults + children	C	1725/1674	-0.41	59%

*R = rhinitis, A = asthma; C = conjunctivitis. **SMD = standardized mean deviation. ***Heterogeneity (very high when I^2^ > 75%).

**Table 2:** **Main characteristics of the 77 randomized controlled trials of SLIT**

**Allergen**	Grass	Dust mite	Parietaria	Ragweed	Others
	34	20	5	5	13
**Duration**	< 6 months	6-12 months	12-24 months	> 24 months	
	31	21	21	4	
**Patients**	<50	51-100	101-200	>200	
	32	21	11	13	

In the last decade, several “large trials” (involving hundreds of patients) were performed and published [4]. These trials provided the evidence and suggested the need for a more uniform design and conduction of clinical studies, in order to harmonize and compare the results obtained, so that robust recommendations can be given. Of note, the methodological problems had been well recognized; therefore recommendations for a better standardization of clinical trials were published and disseminated [15–17].

## Which aspects are relevant to design a slit trial?

### Design

Certainly, it is mandatory that a placebo comparison and a double blind design are used. This is to allow the measurement of the magnitude/size of the effects of SLIT over the non-SLIT group. It has to be kept in mind that, usually, the SLIT trials are not truly placebo controlled (that would be only SLIT versus only placebo), as all patients are allowed to use rescue or regular medications for their symptoms (antihistamines, nasal steroids, bronchodilators etc.). Indeed, SLIT is used as an add-on treatment to rescue medications and it is placebo-controlled in this sense. In addition the placebo effect is usually relevant in immunotherapy trials [18]. Another interesting aspect is that no “equivalent” placebo is available for SLIT, since the local side effects (oral itching, burning, swelling, nausea) cannot be mimicked by a placebo substance. Thus, subjects who are receiving the active treatment in such settings can be quite easily identified by investigators, also considering that the local side effects by the active SLIT usually disappear after 5–10 days [19]. Nonetheless, it is also true that appropriate sub-analyses showed that the possible placebo-unblinding due to local side effects did not affect the validity of results [20]. It is clear that a placebo should at least have the same appearance, taste and smell as the active formulation, but presently it is not feasible to have a totally equivalent placebo [16].

### Patients and exposure

SLIT is allergen-specific, therefore the sensitization to the offending allergen should be clearly demonstrated by the usual standard diagnostic techniques, as well as the causal role of the culprit allergen in inducing symptoms. Monosensitized subjects would be the ideal patients, but in real life the vast majority of patients are polysensitized. On the other hand, it has been shown that, when the causal role of an allergen in inducing symptoms is clearly established and documented, the concomitance of other sensitizations does not affect in general the efficacy of SLIT [21]. Another important aspect is that patients should be symptomatic at the beginning of the study (or during the selection phase or baseline evaluation). If patients have only mild or intermittent symptoms (asthma/rhinitis), an add-on effect is difficult to detect. This was clearly demonstrated by two studies in asthmatic patients [22, 23]. In both studies, no effect of SLIT could be demonstrated in asthma versus baseline, and placebo, but in both studies patients were already optimally controlled by therapy at baseline.

As mentioned above, the exposure to the culprit allergen should be well documented. This is expensive and difficult to apply with dust mite or pet dander, provided that appropriate avoidance measures are applied to all recruited patients. For plant-derived allergens, detailed pollen counts are available almost everywhere. Thus, pollen counts pertinent to the area where the patients live should be provided, and data should be normalized according to pollen count itself (e.g. peak pollen period) for a given area, that may differ in multicenter studies, as shown in recent large trials [24].

### Outcome, sample size, statistical aspects

As happens for traditional drug trials, the primary outcome must be clearly defined, since it defines the sample size and the power of statistical analysis. There is still a debate on the fact that intent to treat (ITT) population should be analyzed. Due to the delayed effects of SLIT, and the need that treatment is assumed for long times, a per protocol analysis also can be justified. All post-hoc analyses must be appropriately declared in the study design [15–17].

Rhinitis symptoms are traditionally measured by a grading that ranges from 0 (absent) to 3 (bothersome) for rhinorrhea, obstruction, sneezing, itching (two or three conjunctival symptoms using the same scale are often added) [25]. Probably, the Visual Analog Scale (VAS) system could replace the traditional categorical scoring [26]. Since symptoms may depend on the use of medications, a combined symptoms + medication score is recommended as the primary outcome [16, 17]. A reduction of that score greater than 20% versus placebo is considered reasonably significant. Recently, an adjusted score considering day-by-day the effect of drugs has been proposed [27].

### Regimens and doses

The regimen of administration and maintenance dose, as described in the literature, largely varies among the different manufacturers. The dose of allergen administered still represents the main obstacle for a well defined harmonization. In fact, each producer standardizes the vaccine according to an in-house reference. The result is that the real amount of allergen(s) contained in each preparation largely varies [28]. In recent years, attempts have been made to have a uniformity in the potency of extracts by expressing the content of allergens in mcg/mL. This has resulted in the ability to perform dose-ranging trials which provided consistent results for grasses in different trials [5, 6], but similar data are still needed for other relevant allergens.

In addition, the administration protocols largely vary among manufacturers. The clinical efficacy of SLIT was demonstrated with tablets, drops or sublingual sprays administered either daily, every other day or twice weekly [29]. For seasonal allergens, although the continuous (all-year long) administration have been shown to be effective also in the long term [24], there is evidence that a pre (-coseasonal) regimen may be equally effective as well [30, 31]. A standardization of doses and administration regimens is urgently needed to clearly assess the efficacy of the treatment and to compare studies and products.

### Safety monitoring

Safety is an essential aspect of SLIT, because it was designed especially to overcome the possible systemic side effects of SCIT as described in several reviews. Nonetheless, the description of safety in clinical trials and post-marketing surveys is variable and sometimes vague, so that a uniform and comprehensive evaluation cannot still be made. It is true that SLIT has, in general, a better safety profile than SCIT [4], but the reporting of adverse events remains largely subjective. In the majority of trials, the traditional EAACI grading system for the severity of systemic side effects was used see [32], although in the case of SLIT, systemic effects are rare, whereas local side effects are common. For these reasons, it has been agreed that the WAO classification for systemic side effects [32] should be used also for SLIT, whereas a specific grading/classification system for local adverse events should be applied only to SLIT [33]. This approach, if universally agreed upon, would allow a more detailed comparison among studies, products and administration regimens.

### Reporting

There is still a large discrepancy between recommendations on how to report clinical trials and, how the reports are actually made, as recommended by the CONSORT guidelines. Unfortunately, this discrepancy is particularly apparent for SLIT and SCIT trials, where the adherence to CONSORT guidelines is poor [34]. Correctly and completely reporting a trial is not simply an academic exercise. A correct reporting allows to better reproduce (or refute) the described results, using a uniform methodology. Finally, since SLIT is self managed by patients, adherence remains a primary concern, but this has been considered only in few trials, and the results reported in experimental settings often differ from the real-life environment [35]. Thus, reporting adherence (doses taken versus prescribed) remains essential in clinical trials, but this aspect is probably more important in real life.

## Conclusion

It is clear, looking at the published articles that SLIT is clinically effective [7]. It is also clear that there is a large heterogeneity among studies, mainly due to the variability in the inclusion criteria, dose of allergen, duration, methodological aspects, and reporting. It is also apparent that studies on allergen immunotherapy require a careful selection of patients and long duration of treatment, possibly in a double blind randomized and controlled fashion. It would be difficult to satisfy all the requirements for a robust study, but some of the possible biases could be corrected, especially by developing a consensus on the outcomes, duration of the trial, criteria for patient selection, and doses to be administered. This latter point remains of great uncertainty, since dose-ranging studies have been performed so far with grass, mite [36] and ragweed [37] allergens. It is also unclear on how early SLIT could be started, and the precise basis for initiating it, although safety data in young children are overall favorable for SLIT. Finally, the assessment of adherence remains a major problem, that is difficult to solve in clinical trials, when the real-life setting is the usual situation.

## Synopsis

The large heterogeneity of clinical trials with SLIT, partially limits the robustness of the evidence provided so far, and also affects meta-analyses. Thus, to correct this bias, a standardization and harmonization of methodology is urgently needed. This concerns in particular the following: the design, outcomes, selection of patients, extracts, description and reporting of adverse events, which are the most variable aspects. Efforts are currently made by the World Allergy Organization and international societies to provide recommendations on how to properly plan and design clinical trials, and how to harmonize outcomes and reporting, in order to make trials comparable each other, progressively reducing the heterogeneity.
